# Osteogenic human MSC-derived extracellular vesicles regulate MSC activity and osteogenic differentiation and promote bone regeneration in a rat calvarial defect model

**DOI:** 10.1186/s13287-024-03639-x

**Published:** 2024-02-07

**Authors:** Niyaz Al-Sharabi, Samih Mohamed-Ahmed, Siddharth Shanbhag, Carina Kampleitner, Rammah Elnour, Shuntaro Yamada, Neha Rana, Even Birkeland, Stefan Tangl, Reinhard Gruber, Kamal Mustafa

**Affiliations:** 1 Department of Clinical Dentistry, Faculty of Medicine, Center for Translational Oral Research (TOR), University of Bergen, 5009 Bergen, Norway; 2 Department of Immunology and Transfusion Medicine, Haukeland University Hospital, 5021 Bergen, Norway; 3 Karl Donath Laboratory for Hard Tissue and Biomaterial Research, University Clinic of Dentistry, Medical University of Vienna, 1090 Vienna, Austria; 4 Ludwig Boltzmann Institute for Traumatology, The Research Center in Cooperation with AUVA, 1200 Vienna, Austria; 5 Austrian Cluster for Tissue Regeneration, 1200 Vienna, Austria; 6 Department of Clinical Medicine, Faculty of Medicine, University of Bergen, 5009 Bergen, Norway; 7 The Proteomics Facility of the University of Bergen (PROBE), University of Bergen, 5021 Bergen, Norway; 8 Department of Oral Biology, University Clinic of Dentistry, Medical University of Vienna, 1090 Vienna, Austria; 9 Department of Periodontology, School of Dental Medicine, University of Bern, 3010 Bern, Switzerland

**Keywords:** Extracellular vesicles, Mesenchymal stem cells, Naïve-EVs, Osteo-EVs, Bone regeneration, Rat calvarial defects

## Abstract

**Background:**

There is growing evidence that extracellular vesicles (EVs) play a crucial role in the paracrine mechanisms of transplanted human mesenchymal stem cells (hMSCs). Little is known, however, about the influence of microenvironmental stimuli on the osteogenic effects of EVs. This study aimed to investigate the properties and functions of EVs derived from undifferentiated hMSC (Naïve-EVs) and hMSC during the early stage of osteogenesis (Osteo-EVs). A further aim was to assess the osteoinductive potential of Osteo-EVs for bone regeneration in rat calvarial defects.

**Methods:**

EVs from both groups were isolated using size-exclusion chromatography and characterized by size distribution, morphology, flow cytometry analysis and proteome profiling. The effects of EVs (10 µg/ml) on the proliferation, migration, and osteogenic differentiation of cultured hMSC were evaluated. Osteo-EVs (50 µg) or serum-free medium (SFM, control) were combined with collagen membrane scaffold (MEM) to repair critical-sized calvarial bone defects in male Lewis rats and the efficacy was assessed using µCT, histology and histomorphometry.

**Results:**

Although Osteo- and Naïve-EVs have similar characteristics, proteomic analysis revealed an enrichment of bone-related proteins in Osteo-EVs. Both groups enhance cultured hMSC proliferation and migration, but Osteo-EVs demonstrate greater efficacy in promoting in vitro osteogenic differentiation, as evidenced by increased expression of osteogenesis-related genes, and higher calcium deposition. In rat calvarial defects, MEM with Osteo-EVs led to greater and more consistent bone regeneration than MEM loaded with SFM.

**Conclusions:**

This study discloses differences in the protein profile and functional effects of EVs obtained from naïve hMSC and hMSC during the early stage of osteogenesis, using different methods. The significant protein profile and cellular function of EVs derived from hMSC during the early stage of osteogenesis were further verified by a calvarial bone defect model, emphasizing the importance of using differentiated MSC to produce EVs for bone therapeutics.

**Supplementary Information:**

The online version contains supplementary material available at 10.1186/s13287-024-03639-x.

## Introduction

Bone tissue is a dynamic, intricate structure which undergoes continuous remodelling throughout an individual's lifetime. Disorders such as fractures and abnormalities can disrupt the normal bone-healing process, causing long-term morbidity and impairment. Current treatment approaches for bone healing include bone grafts and synthetic biomaterials. However, these techniques are not always effective and may lead to complications such as graft failure, infection, and immunological rejection [1]. There is increasing interest in the clinical potential of tissue engineering strategies using hMSC, biomaterials, and growth factors for bone regeneration [2]. While MSC have shown the ability to regenerate various tissues, the functional improvements observed after implantation do not always correlate with the number of MSC detected in situ [3]. This suggests that the secretome of MSC, which contains bioactive molecules such as cytokines, chemokines, growth factors and extracellular vesicles (EVs), such as microvesicles and exosomes, may modulate the effects of MSC on tissue regeneration through paracrine pathways [4].

The composition of MSC secretome or conditioned medium (MSC-CM) is highly dependent on the cell source and can be altered by various stimuli, such as hypoxia, cytokines, and serum deprivation. Several studies have shown that CM derived from MSC under different conditions can effectively promote bone healing and regeneration [5, 6]. EVs are among the key factors for these therapeutic effects. EVs are small vesicles (30–1000 nm in diameter) enclosed by a membrane, enriched with bioactive molecules such as lipids, proteins, and microRNAs [7]. While EVs isolated from MSC are reported to trigger bone healing [8], their therapeutic potential may vary according to the cellular origin, degree of differentiation and culture conditions. In recent years, there has been increasing interest in harnessing the therapeutic potential of EVs or exosomes to increase the osteogenic activity of MSC. Various studies have explored different strategies to achieve this, including exposing cultured MSC to osteogenesis-promoting factors or overexpressing osteogenic genes [8, 9]. Research has shown that exosomes released from MSC under osteogenic induction medium (OM) can upregulate genes related to osteogenesis and bone mineralization in vitro, indicating their potential as a therapeutic tool [9]. EVs derived from MSC treated with osteogenic media at different time points were found to be enriched with certain proteins and miRNAs with osteogenic properties, highlighting their potential as a source of therapeutic agents [10]. EVs derived from chemically or genetically osteogenically induced differentiated hMSC have been shown to induce the differentiation of undifferentiated MSC into osteoblasts in vitro [11]. This suggests that these EVs have remarkable osteogenic properties, which can be exploited for therapeutic purposes. Moreover, EVs released from hMSC during the late stage of osteogenic induction have shown significant potential in promoting osteogenic differentiation of undifferentiated MSC, further emphasizing their therapeutic potential [12]. However, isolating EVs or exosomes from the late stage of osteogenesis can be challenging, due to the disruption of their membrane structure caused by hydroxyapatite crystal formation [13]. Furthermore, EVs derived from mineralized primary osteoblasts have been observed to undergo changes in morphology and proteome profiles during in vitro osteogenesis, particularly in the late stage [14]. This highlights the importance of understanding the complex interplay between EVs and the osteogenic microenvironment. In this context, it is of interest to note that in a recent study, exosomes released from MSC during the early stage of osteogenesis promoted bone formation in mice with calvarial bone defects, highlighting the potential of early-stage EVs as a therapeutic tool [8].

A common clinical approach for treating advanced bone defects is guided bone regeneration (GBR), using occlusive barrier membranes, such as bioabsorbable collagen membranes (MEM) [15, 16]. These membranes function as a biocompatible barrier, which prevents the infiltration of competing soft tissue cells [17] and has the potential to serve as a carrier for various bioactive macromolecules, making them a promising delivery agent for bone formation applications [18]. We have recently reported the efficacy of MEM functionalized with hMSC-CM in rat calvarial bone defects, achieving bone formation comparable with that of implanted rat MSC [19]. It may be hypothesized that incorporating EVs into MEM can enable their sustained release and potentially improve the therapeutic efficacy of cell-free strategies for bone regeneration [20].

Despite the remarkable potential of EVs derived from undifferentiated or "naïve" MSC as an acellular therapy for bone defects, there remains a significant gap in knowledge regarding the properties and functions of EVs sourced from osteogenic differentiated MSC. In harvesting EVs, it is crucial to select carefully and expand a homogeneous population of cells with a known differentiation status: this is essential for obtaining reproducible results and producing EVs with predictable biological properties [14, 21]. Addressing these knowledge gaps is vital to achieving the full potential of acellular therapies using osteogenic EVs. The objectives of this study were therefore to investigate (a) the proteomic profile of EVs derived from naïve hMSC (Naïve-EVs) and MSC at an early stage of osteogenesis (Osteo-EVs), (b) the capacity of Osteo-EVs to promote in vitro hMSC proliferation, migration, and osteogenic differentiation compared with Naïve-EVs and (c) the capacity of Osteo-EVs to enhance in vivo bone formation in critical-sized rat calvarial defects. These investigations are intended to advance our knowledge of Osteo-EVs, with special reference to their potential as a novel acellular therapy for bone defects.

## Materials and methods

### Cell culture

The bone marrow mesenchymal stem (hMSC) used in this study was isolated from the anterior iliac crest of healthy human donors after ethical approval from the Regional Committee for Medical and Health Research Ethics in Norway (2013/1248/REK sør-øst C). The cells were expanded in growth medium (GM, Dulbecco’s Modified Eagle’s Medium (DMEM, Invitrogen, Carlsbad, CA, USA) supplemented with 1% penicillin/streptomycin (GE Healthcare, South Logan, UT, USA) and 10% foetal bovine serum (FBS; GE Healthcare) at 37 °C in a humidified atmosphere, with 5% CO_2_. The medium was replaced twice a week. Based on the minimal criteria proposed by the International Society for Cellular Therapy (ISCT) to define hMSC, the cells were characterized for negative expression of surface marker antigens: CD34, HLA-DR, and CD45, and positive expression of surface marker antigens; CD73, CD90, and CD105, using flow cytometry. The cells were then tested for their multi-lineage differentiation potential into osteogenic, adipogenic, and chondrogenic lineages, using Alizarin Red S, Oil Red O, and Alican Blue staining methods, respectively, as described previously [22].

### Preparation of osteogenic- and Naïve-conditioned media

To collect the conditioned medium (CM) from osteogenic and naïve (non-induced) culture conditions, at passages 3–5, hMSC from donors (*n* = 3) was trypsinized and seeded at 4000 cells/cm^2^ in GM. To generate CM from hMSC undergoing osteogenic differentiation, the cells were cultured in the osteogenic induction medium (OM) containing 100 nM dexamethasone, 45 μM ascorbic acid and 20 mM β-glycerophosphate (all from Sigma-Aldrich, St. Louis, MO, USA). The cells were maintained in this medium for 7 days. Next, the cells were washed three times with pre-warmed PBS and cultured in a serum-free medium (SFM), i.e. DMEM without supplements, for 3 days at 37 °C in 5% CO_2_, replaced with fresh SFM after 2 days. CM collected from the first 2 days and the third day was pooled and defined as an osteogenic-conditioned medium (Osteo-CM). To achieve a non-induced condition, after 7 days of incubation, the cells were cultured in SFM for 3 days at 37 °C in 5% CO_2_ and replaced after 2 days. After 3 days of incubation of cells in SFM, CM collected from the first 2 days and the third day was pooled and defined as a Naïve-conditioned medium (Naïve-CM). At the end of the experiment, collected CM was centrifuged at 4 °C (3000′ g for 5 min, followed by 2000′ g for 10 min) to remove cell debris and apoptotic bodies, followed by filtration through an 0.2-µm filter to remove larger particles. The prepared CM samples were then stored at − 80 °C.

### Isolation of EVs

Size-exclusion chromatography (SEC) was used to isolate EVs from each group [23]. Briefly, Osteo- and Naïve-CM were concentrated using Amicon Ultra-15 Centrifugal Filter Units with Ultracel-100 membrane (MWCO = 100 kDa; Merck Millipore, USA) to ≤ 300 µl by repeated centrifugation at 3600 × g. The concentrate was adjusted to 500 µL using 0.2 µm-filtered and degassed PBS (dPBS), collected, and stored at − 80 °C as concentrated CM (CCM). Next, to isolate EVs from Naïve-CM (Naïve-EVs) and from Osteo-CM (Osteo-EVs), 500 µl of each respective CCM was applied to a 10-ml qEV column (35 nm qEVoriginal, no. 1000871, Izon Science Ltd) after columns had been equilibrated with 10 ml of PBS, as recommended by the manufacturer. The respective CCM was then pipetted onto the column to collect the output fractions. The first 3 ml (1st–6th fractions) was discarded as non-EV flowthrough, while the second 2.5 ml (7th–11th fractions) was collected as EV fractions and stored at – 80 °C until further analysis.

### Characterization of Osteo- and Naïve-EVs

The immunophenotype of Osteo- and Naïve-EVs was analysed using immuno-affinity-based Dynabeads® magnetic separation technology via the flow cytometry method, to confirm the presence of EV-specific tetraspanin markers CD63, CD81, and CD9 (BD Bioscience, USA) according to the manufacturer’s recommendations (Life Technologies—Invitrogen, USA). Briefly, 10 µg of EVs from each group was resuspended in 1% BSA prepared in PBS and labelled with 20 µl of beads (Life Technologies—Invitrogen, USA) specific for each antibody (CD81, CD9, and CD63) by overnight incubation with gentle agitation. The solutions containing bead-bound EVs (EVs-beads) were then centrifuged and washed twice with 0.1% BSA. Finally, the EV beads were incubated with PE-labelled anti-human antibodies; CD63, CD81, and CD9, along with purified mouse IgG1, κ isotype control (BD Bioscience, USA) for 60 min at room temperature (RT). Data were acquired using an Accuri6 flow cytometer (BD Biosciences, Franklin Lakes, NJ, USA) and analysed using FlowJo software (FlowJo V10.6.2). The size distribution of EVs was determined using dynamic light scattering (DLS, Zetasizer system, Malvern, UK). The protein concentration of EV samples was measured by Pierce™ BCA Protein assay kit (Thermo Fisher Scientific, USA), following the manufacturer’s instructions. EVs morphology was recorded by transmission electron microscopy (TEM), according to a previously described protocol [24]. The final EV preparations were examined using a Jeol JEM1400 transmission electron microscope (Jeol Ltd., Tokyo, Japan).

### Liquid chromatography with tandem mass spectrometry (LC–MS/MS)

#### *Sample preparation*

Protein lysates collected from Osteo- and Naïve-EVs (*n* = 3 each) were analysed using LC–MS/MS with Label-Free Quantitation [25, 26]. In brief, approximately 25 μg of protein was digested into tryptic peptides. About 0.5 µg protein dissolved in 2% acetonitrile and 0.5% formic acid was injected into an Ultimate 3000 RSLC system connected online to an Exploris 480 mass spectrometer equipped with EASY-spray nano-electrospray ion source (all from Thermo Scientific, Sunnyvale, CA, USA) [26].

#### *Bioinformatic analysis*

LC–MS/MS raw data were searched using Proteome Discoverer software (version 435 2.5.0.400; Thermo Scientific). The Osteo- and Naïve-EVs protein datasets were then filtered and analysed for relative protein quantification among EV donors, using Perseus version 2.0.3.1 software. To ensure the precise quantification of proteins in each EV group, proteins that were not present in all three donors of each EV group were filtered out. The Venn diagram was used to compare the presence of common EV proteins in each EV group with the Extracellular vesicles database (including the top_100 proteins from Vesiclepedia data, http://microvesicles.org/) [27]. To identify the Gene Ontology Biological Process (GOBP), Gene Ontology Molecular Function (GOMF), and Gene Ontology Cellular Components (GOCC) associated with the common EV proteins of each EV group, a FunRich analysis tool (Version 3.1.3) was used. For statistical analysis, LIMMA was used (https://www.ncbi.nlm.nih.gov/pmc/articles/PMC4402510/) and DEqMS packages in R (v.4.2.1) (https://pubmed.ncbi.nlm.nih.gov/32205417/) to investigate differentially expressed proteins (DEPs) between Osteo- and Naïve-EVs. To predict GOBP, GOMF and Reactome pathways (REC) of DEPs in Osteo- and Naïve-EVs datasets, we used the Gene Ontology Resource version 17.0 (Panther) using Fisher's exact test as a type and false discovery rate (FDR) as a correction method [28]. To predict the potential participation of EV proteins in bone-related biological processes, we retrieved relevant bone biological process terms from QuickGO (https://www.ebi.ac.uk/QuickGO/, accessed in December 2022, EMBL-EMI, Cambridge, United Kingdom) and compared them with the names of DEPs and unique EV proteins (proteins not included in the statistical analysis) between Osteo- and Naïve-EVs.

### In vitro osteogenic potential of EVs

#### *Cell uptake*

To determine the internalization of Osteo- and Naïve-EVs by the hMSC, 10 µg of Osteo- and Naïve-EVs, respectively, was labelled with a green fluorescent lipophilic dye- 3 mM DiO'; DiOC18 [3] (3,3′-Dioctadecyloxacarbocyanine Perchlorate/DMSO (DiOC18, Invitrogen™) for 2 h at 37 °C by gentle rotation [29]. Non-binding DiOC18 dye was then removed by diluting samples with PBS, followed by centrifugation using Vivaspin® 2, 100 kDa MWCO Polyethersulfone filters (Sartorius Stedim Biotech GmbH) at 300 × g for 3 min at 4 °C. hMSC was then washed twice with PBS and incubated at 37 °C with labelled EVs for 48 h, before fixing with 4% paraformaldehyde for 20 min. For staining of filamentous actin (F-actin), phalloidin–tetramethylrhodamine B isothiocyanate (Sigma-Aldrich, St. Louis, MO, USA) was added and incubated for 20 min at room temperature. Cellular nuclear DNA was then stained with 4′,6-diamidino-2-phenylindole (DAPI) for an additional 20 min. The cellular uptake of EVs was imaged using a Dragonfly 505 confocal spinning disc system (Andor Technologies, Inc, Belfast, Northern Ireland).

#### *Cell proliferation*

An Alamar blue cell viability assay (Thermo Fisher Scientific, Eugene, USA) was conducted after 24 and 72 h to assay cell proliferation. Briefly, hMSC was seeded at a density of 2 × 10^3^ cells/100 μl of GM in a 96-well plate and cultured at 37 °C in 5% CO_2_ for 48 h. The hMSC was then washed with PBS and exposed to DMEM supplemented with 10% exosome-depleted FBS (FBS-ED-12F, Capricorn Scientific GmbH, Germany) and 10 μg/ml of Osteo- or Naïve-EVs, respectively, were added. All treatments were performed on day 1 (48 h after cell adhesion) and day 4 (72 h after the first dose). Next, 90 μl of fresh medium was mixed with 10 μl Alamar blue and incubated for 3 h at 37 °C with 5% CO_2_. The reduction of Alamar blue solution was measured by fluorescence values at 560 nm (excitation) and 590 nm (emission) using a Varioskan LUX Multimode Microplate Reader (Thermo Fisher Scientific, Vantaa, Finland). Cell viability was assessed according to the following formula: Viability (%) = OD of experimental group/OD of control medium group × 100.

#### *Cell migration*

The effect of Osteo- and Naïve-EVs on MSC migration was evaluated by an in vitro wound-healing assay. Briefly, hMSC was seeded in a culture-insert (ibidi culture-insert 2 well, ibidi GmbH, Martinsried, Germany) at a density of 3 × 10^4^ cells per well. When the cells reached over 100% confluence, the culture-insert was removed, and the cells were washed with PBS to remove non-adherent cells. To stop cell proliferation, 20 µg/ml of Actinomycin D-Ready Made Solution (Sigma-Aldrich, St. Louis, MO, US) was added and incubated at 37 °C in 5% CO_2_ for 2 h. The cells were washed with PBS and then different EV treatment groups were added. The hMSC was photographed at time points (*t*); *t* = 0 h, 24 and 48 h, in an inverted microscope (Eclipse TS100, Nikon, Tokyo, Japan). To track the wound closure, lines along the leading edges of each cell front were made, followed by measuring the decrease in the average distances of the lines using ImageJ software (version 1.52) [30]. The migration area was assessed using the following equation: migration area (%) = (*A*_0_ – *A*_*f*_)/*A*_0_ × 100, where *A*_0_ represents the initial wound area (*t* = 0 h) and *A*_*f*_ represents the residual area of the wound at *t* = 24 h and 48 h, respectively [31].

#### *Osteogenic differentiation*

To study the effect of the different EVs on the osteogenesis of cultured hMSC, 10 µg/ml of Naïve- or Osteo-EVs was added to the OM. OM supplemented with an equal volume of PBS served as a control medium group. The media were changed twice a week, and EVs were added each time. After 14 days, alkaline phosphate staining (ALP) activity was tested using a BCIP/NBT alkaline phosphatase colour development kit, following the manufacturer’s instructions (Sigma-Aldrich, St. Louis, MO, MUSA). After 21 days, calcium nodule formation was assessed by staining with 2% Alizarin Red Staining (ARS) (Sigma-Aldrich, St. Louis, MO, USA). Images were taken using a digital camera (ECLIPSE TS100, Nikon, Tokyo, Japan). To quantify calcium deposition, 10 mM% cetylpyridiniunm chloride (Sigma-Aldrich, St. Louis, MO, USA) was added and incubated for 20 min. Absorbance was then measured at 540 nm using a Varioskan LUX Multimode Microplate Reader (Thermo Fisher Scientific, Vantaa, Finland). To determine the effect of the Osteo- and Naïve-EVs on the expression of osteogenesis-related genes in hMSC after 14 days of incubation, an osteogenic gene expression array and the validation of individual genes by qRT-PCR were conducted. Briefly, total RNA was extracted from hMSC using a Maxwell® 16 LEV simply RNA kit (Promega, Madison, WI, USA). RNA amount and purity were measured using Nanodrop ND-1000 Spectrophotometer (Nanodrop Technologies, Wilmington, DE, USA). cDNA was synthesized using a High-Capacity cDNA Reverse Transcription Kit (Applied Biosystems, Foster City, CA, USA). The gene expression array (4,413,255, Applied Biosystems) and RT-qPCR, using TaqMan Fast Universal PCR Master Mix (Applied Biosystems), were assessed using a Step one™ Real-Time PCR System (Applied Biosystems). The array (4,413,255, Applied Biosystems, USA) was tailor-made (96 genes), covering, but not limited to, putative osteogenic differentiation markers, bone extracellular matrices, and TGF/BMP signalling pathways. The set of targeted genes and CT values are presented in Additional file 1. The expression levels were normalized by the following housekeeping genes: RNA, 18S ribosomal 1 (18S rRNA), Glyceraldehyde-3-Phosphate Dehydrogenase (GAPDH), Hypoxanthine guanine phosphoribosyltransferase 1 (HPRT1) and beta-glucuronidase (GUSB). The expression of the osteogenesis-related human genes runt-related transcription factor 2 (RUNX2), collagen type I (Col 1A2), bone morphogenetic protein 2 (BMP-2), alkaline phosphatase (ALPL), osteopontin (SPP1), bone sialoprotein (BSP) and osteocalcin (BGLAP), was validated by real-time quantitative reverse transcription (RT-qPCR). The human glyceraldehyde-3-phosphate dehydrogenase (GAPDH) gene served as an endogenous control. The comparative Ct method was used for the relative measurement of gene expression level against the GAPDH gene. As shown in Additional file 2, all primers were from Applied Biosystems.

### Effect of Osteo-EVs in vivo

#### *Functionalization of MEM*

To evaluate the effectiveness of MEM loaded with Osteo-EVs, on bone repair in vivo*,* a bilayer, non-cross-linked collagen membrane (25 mm × 25 mm; Bio‐Gide®, Geistlich Pharma, Wolhusen, Switzerland) was used. Briefly, MEMs were cut into small pieces (7 mm × 6 mm) using sterile scissors and placed with the “dense/smooth” surface facing the culture surface of the 48 -well plates and the “rough” surface facing upwards. Approximately 2.7 × 10^10^ particles of Osteo-EVs (50 µg in each 170 µl dPBS), or SFM as a control, were loaded onto each membrane and stored under sterile conditions at 4 °C for at least 4 h, to allow the Osteo-EVs to be absorbed [32]. The supernatants were then aspirated, and both Osteo-EVs- and SFM-loaded MEMs were stored at − 80 °C for lyophilization after adding a freeze–thaw protective agent mannitol (Sigma-Aldrich, St. Louis, MO, USA) at a final concentration of 0.5% (w/v). On the day of the animal experiments, lyophilized Osteo-EVs- and SFM-loaded MEMs were thawed and stored under sterile conditions at 4 °C until implantation.

#### *Characterization of Osteo-EV-functionalized MEM*

To evaluate the overall distribution of Osteo-EVs on MEM, Osteo-EVs- (50 ug/MEM) and SFM-loaded MEM were fixed with 2.5% glutaraldehyde in PBS for 30 min. Non-functionalized MEM served as controls. The fixed MEMs were washed three times with PBS and then dehydrated in an ascending series of ethanol. After evaporation of the ethanol, the samples were dried at room temperature, sputter-coated with gold–palladium and viewed in a scanning electron microscope (SEM) at 5 kV with a ZEISS SUPRA 55 VP. The average size of the Osteo-EVs in the MEM was determined using ImageJ 1.54 g.

#### *Release of Osteo-EVs from MEM and uptake by cultured hMSC*

The release of Osteo-EVs from MEMs and their uptake by hMSC were studied in vitro. Briefly, labelled Osteo-EVs/MEMs (10 µg) were positioned with the “rough” surface towards cultured hMSC in 8-ibidi wells (Nunc® Lab-Tek® Chamber Slide™ system). After 48 h, the Osteo-EVs/MEMs were removed and the cultured hMSC were washed twice with PBS, fixed with 4% paraformaldehyde for 15 min and stained with phalloidin–tetramethylrhodamine B isothiocyanate (Sigma-Aldrich, St. Louis, MO, USA) and DAPI as described above. The cells were then viewed in a Dragonfly 505 confocal spinning disc confocal scanning microscope (Andor Technologies, Inc, Belfast, Northern Ireland).

#### *Calvarial defect model*

All procedures were approved by the Norwegian Animal Research Authority (Mattilsynet; FOTS-17443) and conducted following the ARRIVE guidelines. Five male Lewis rats (LEW/OrlRj, Janvier Labs, Le Genest-Saint-Isle, France), 7 weeks old and weighing 200–250 g, were acclimated to standard vivarium conditions for two weeks. Before surgery, the animals were anaesthetized with a mixture of sevoflurane (Abbott Laboratories, Berkshire, UK) and O_2_ using a custom-made mask. As described previously [33], a 2-cm sagittal incision was made in the midline of the cranium and the periosteum was reflected to expose the parietal bones. Using a 5-mm outer-diameter trephine bur (Meisinger GmbH, Neuss, Germany), two full-thickness defects were created in each animal, one in each parietal bone. MEM loaded with Osteo-EVs (*n* = 5) or SFM (*n* = 5) as control was then randomly applied to the defects. The membranes were positioned with the “rough” surface towards the calvaria/defect (“dense/smooth” surface towards the soft tissue) and stabilized by applying ~ 5 μL of tissue adhesive (Histoacryl; B. Braun, Tuttlingen, Germany) at the corners. The rats were coded via ear clips following specific randomization. The allocation of treatments was adapted such that each animal received both treatments. After 2 weeks, an in vivo micro-computed tomography (µCT) scan was performed. At 4 weeks, the rats were euthanized with an overdose of CO2 and the calvaria was harvested and fixed in 4% paraformaldehyde. The primary outcome of interest was the new bone formation after 2 weeks using in vivo µCT and 4 weeks using ex vivo µCT and histology. For all experimental steps involving handling/analyses, animals and samples were identified by numbers to facilitate operators' blinding of treatment groups.

#### *Micro-CT analysis*

To follow the in vivo bone regeneration, the calvaria of the live rats was scanned 2 weeks after surgery by computed tomography (in vivo CT) under general anaesthesia, using a small-animal CT scanner and Mediso workstation (NanoScan Mediso, Budapest, Hungary): voxel size 40 μm, energy 70 kV, exposure 32 times 300 ms, projections 720, and 1:1 binning. For ex vivo μCT analysis, harvested samples were scanned using a SCANCO 50 μCT scanner (SCANCO Medical AG, Bruttisellen, Switzerland) at 90 kV and 200 μA, with an isotropic resolution of 20 μm. Both in vivo CT and ex vivo μCT scans were reconstructed using Amira software (Thermo Scientific) by orienting the drill direction along the Z-axis, with the defect in the approximate centre of the image. A standardized density threshold and volume of interest—including the entire thickness of the calvaria and excluding 0.5 mm of marginal bone—was defined for each defect. The percentage of new bone formation was calculated as a ratio of the total defect volume (BV/TV%). Bone coverage was calculated using ImageJ software (NIH) with custom-defined rulesets for CT and µCT scans.

#### *Histology and histomorphometry*

To visualize newly formed bone and soft tissues in the harvested samples 4 weeks after implantation, standardized thin-ground sections were prepared in the centre of each defect, parallel to the sagittal suture and perpendicular to the parietal bone. The samples were first dehydrated in an ascending series of alcohol and embedded in photocuring resin (Technovit 7200 + 1% benzoyl peroxide, Kulzer & Co., Wehrheim, Germany) before further processing using the EXAKT cutting and grinding equipment (EXAKT Apparatebau, Norderstedt, Germany). Undecalcified thin-ground sections were reduced to a thickness of approximately 100 µm and stained with Levi-Lazko dye (Morphisto GmbH, Frankfurt, Germany). The slices were scanned using an Olympus BX61VS digital virtual microscopy system (DotSlide 2.4, Olympus), with a 20 × magnification at a resolution of 0.32 μm per pixel. For histomorphometric analysis, respective areas of new bone without embedded MEM fibres (defined as new bone), new bone with embedded MEM fibres (defined as hybrid bone), total new bone (sum of new and hybrid bone), mineralized MEM fibres, residual MEM (non-mineralized MEM fibres), indefinite mineralized areas (defined as non-specific mineralization), which could not be attributed to any other category and soft tissue, were manually segmented and quantified in the central defect region using Adobe Photoshop (version 2022, Adobe, San Jose, CA, USA). Corresponding percentages of each tissue type were calculated as a ratio of the total central defect area. The central defect region was limited superiorly by the MEM, inferiorly by the dura mater and laterally by the defect edges.

### Statistical analysis

All acquired data were tested by three donors and presented as the mean ± standard deviation. Multiple group comparisons were conducted by one-way ANOVA. Differences between the two groups were tested by Student’s t-test (normal distribution). Statistical analysis was undertaken using Graph Pad Prism 9.3.1 software (GraphPad, San Diego, CA, USA), and *p* < 0.05 was considered statistically significant.

## Results

### Isolation and characterization of hMSC and obtained EVs

**Fig. 1:**
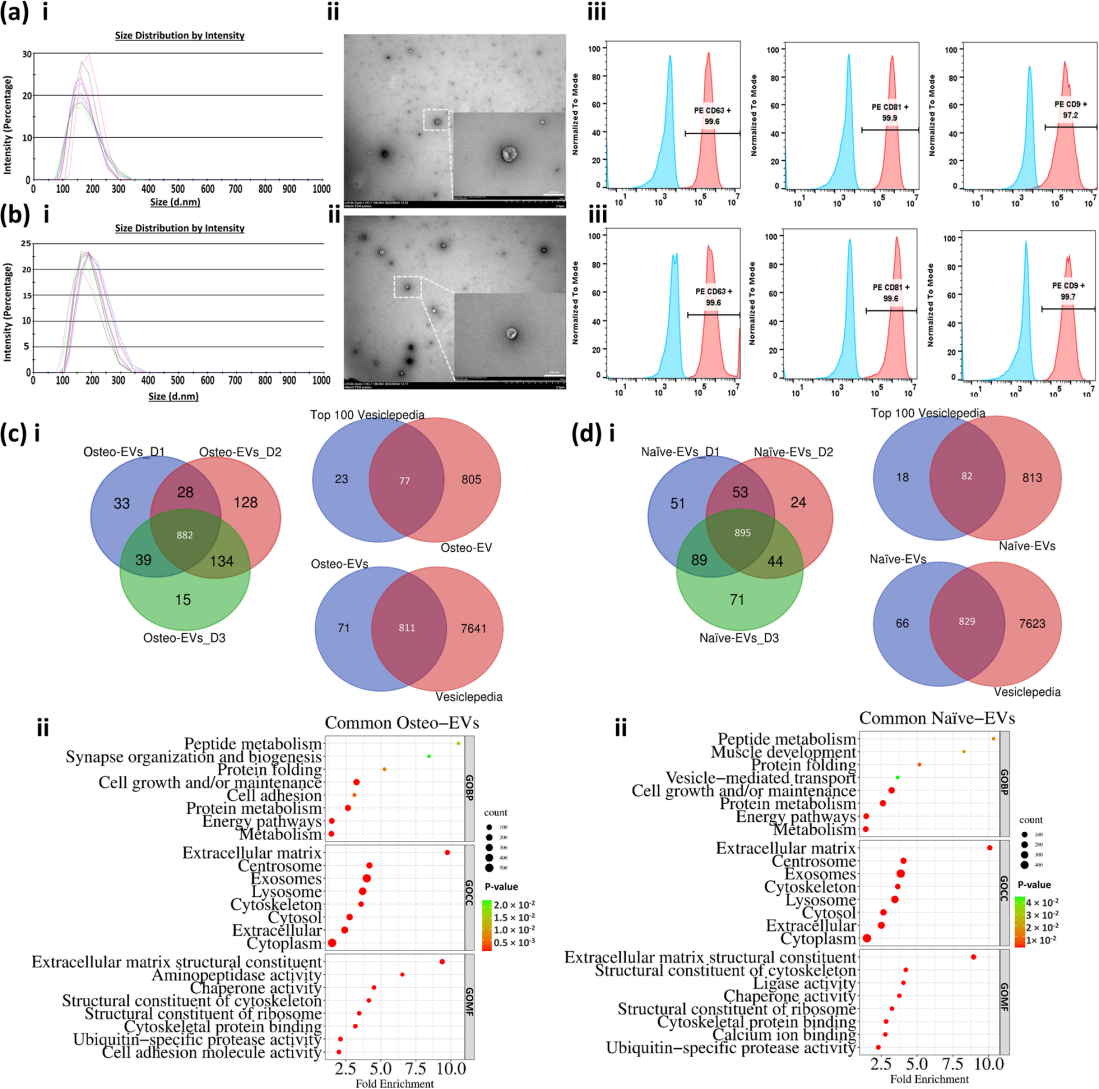
Characterization of Osteo- and Naïve-EVs. **a i** and** b i** Histogram graphs depicting the average size of EVs particles (*n* = 3) in Osteo- and Naïve-EVs, measured by dynamic light scattering (DLS). **a ii** and **b ii** Representative transmission electron microscopy (TEM) images of Osteo-and Naïve-EVs (Magnification of the EVs in the main image x10k; scale bar = 2 µm, and in the small box x70K; scale bar = 200 nm). **a iii** and **b iii** Representative flow cytometry analysis of transmembrane proteins CD63, CD81, and CD9. **b i** Venn diagram across all 3 donors in the Osteo-EVs group and inter-donor comparison, and across the common Osteo-EV (882) proteins and both Vesiclepedia Top 100 proteins and Vesiclepedia datasets. **c ii** The enriched GO terms (BP, CC, and MF) for the common Osteo-EVs (882) proteins using FunRich tool (Version 3.1.3). **d i** Venn diagram across all 3 donors in the Naïve-EVs group and compared to one another, and across the common Naïve-EV (895) proteins and both Vesiclepedia Top 100 proteins and Vesiclepedia datasets. **d ii** The enriched GO terms (BP, CC, and MF) for the common Naïve-EVs (895) proteins using FunRich tool (Version 3.1.3)

hMSC was isolated and characterized based on immunophenotype and multi-differentiation capacity, according to the minimal criteria for defining hMSC, as proposed by ISCT [22, 34]. The isolated and collected Osteo- and Naïve-EVs were characterized. The average diameter of the Osteo-EVs particles (*n* = 3) was 162.8 nm (± 35.25 nm) (Fig. 1a i). The corresponding value for Naïve-EVs (*n* = 3) was 185 nm (± 41.76 nm) (Fig. 1b i). As observed by TEM, the Osteo- and Naïve-EVs displayed a typical cup-shaped vesicle (Fig. 1a, b, ii). Flow cytometry results confirmed the presence of EV surface markers CD63, CD81 and CD9 (> 95% each) (Fig. 1a, b, iii).

### Proteomic analysis of Osteo- and Naïve-EVs

#### *Horizontal bioinformatic analysis of the three donors of Osteo- and Naïve-EV groups*

The proteomes of donor-matched Osteo- and Naïve-EVs from three independent donor-sample preparations were compared, using a label-free LC–MS/MS approach. The total number of proteins quantified for each of the two EV groups was similar: 1259 proteins in the Osteo-EVs and 1227 in the Naïve-EVs (Additional file 1). A Venn diagram was first used to analyse the common and different EV proteins of each EV group derived from three donors, D1, D2, and D3. In the Osteo-EVs group, 982, 1172 and 1070 proteins were identified in each respective donor: there were 882 common proteins, present in all three samples (Fig. 1c i). Among these 882 common proteins, 77 matched the typical Vesiclepedia top-100 markers, while 811 matched the whole EVs/exosome proteins in the Vesiclepedia database (Fig. 1c i). In the Naïve-EVs group, 1088, 1016, and 1099 proteins, respectively, were identified in each donor: there were 895 common proteins, present in all three samples (Fig. 1d i), of which 82 matched the typical Vesiclepedia top-100 markers and 829 matched the whole EVs/exosome proteins in the Vesiclepedia database, as shown in Fig. 1d i. Using the FunRich tool, Gene ontology enrichment analysis was used to analyse the common Osteo-EV (882) proteins and Naïve-EV (895) proteins involved in the GOBP, GOCC and GOMF terms (Fig. 1c, d, ii). The results showed that both common Osteo- and Naïve-EV proteins were related mainly to several GOCC, including “extracellular matrix, “centrosome”, “exosome”, “lysosomes”, and “cytoplasm”. Concerning GOMF, proteins of both EV groups were related to various similar terms, such as “Extracellular matrix structural constituent”, “Structural constituent of cytoskeleton”, “Chaperone and Ubiquitin-specific protease activities” and “Cytoskeletal protein binding”. For GOBP, the terms “Cell growth and/or maintenance”, “Protein metabolism”, “Energy pathways”, “Metabolism”, “Protein folding” and “protein metabolism,” were enriched in both EV groups. “Muscle development” and “Vesicle-mediated transport” were enriched only in the common Naïve-EVs group of proteins, while “Cell adhesion” and “Synapse organization and biogenesis” were enriched in the common Osteo-EV group of proteins.

#### *Horizontal bioinformatics analysis of the Osteo- and Naïve-EV groups*

**Fig. 2:**
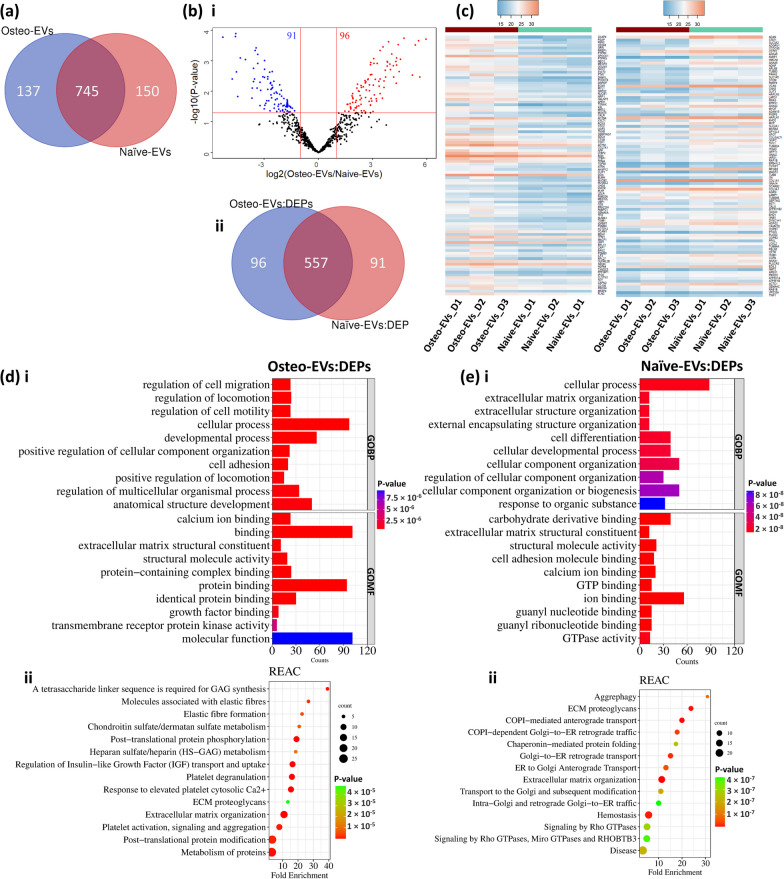
Analysis of DEPs in proteins (745) common to Osteo- and Naïve-EVs groups. **a** Venn diagram representing the numbers of unique and overlapping proteins among the common protein in each EV group. **b i** and **ii** Volcano plot and Venn diagram showing differentially expressed proteins (*p* < 0.05) when comparing Osteo-EVs to Naïve-EVs. **c** Heat map showing the distribution of DEPs among the three donors of each EV group. Values represent protein level quantification (log2). **d** and** e** The enriched GO terms (BP, and MF) and REAC pathways for the DEPs in each EV group, respectively, are retrieved from the gene ontology resource

A comparison of the proteins in the Osteo- and Naïve-EV groups (882 and 895, respectively) disclosed a total of 745 proteins common to both groups and 137 and 150 proteins, respectively, unique to the Osteo- and Naïve-EVs groups (Fig. 2a). These data are all presented in Additional file 1. The DEqMS method was then used for protein level differential analysis and to compare Osteo-EVs to Naïve-EVs (745 common proteins). This analysis resulted in the identification of a total of 187 DEPs: 96 upregulated in the Osteo-EVs group and 91 downregulated compared to Naïve-EVs (Volcano plot and Venn diagram, Fig. 2b i and ii). The data of all DEPs in each EV group are presented in Additional file 1. The proteome differences between the Osteo- and Naïve-EVs of all DEPs are visualized as a heatmap (Fig. 2c). To provide an overview of the GO annotation terms (BP, and MF) and the Reactome pathways of DEPs proteins among Osteo- and Naïve-EVs groups, the gene ontology resource Panther 17.0 was used for analysis [28]. The results showed that DEPs in the Osteo-EVs group were related mainly to several GOBP terms, of which “regulation of cell migration”, “regulation of cell motility and locomotion” and “developmental and cellular processes” were the most enriched processes (Fig. 2d i). In terms of GOMF, “binding and activity” functions were the most enriched terms. This includes “calcium ion binding, “protein binding”, “protein-containing complex binding”, and “growth factor binding” (Fig. 2d i). In terms of the REAC pathway, several pathways were enriched, including but not limited to “extracellular matrix organization”, “post-translational protein phosphorylation”, “platelet degranulation”, “regulation of Insulin-like Growth Factor (IGF) transport and uptake”, “elastic fibre formation”, “chondroitin sulfate/dermatan sulfate metabolism” and “Heparan Sulfate/Heparin (HS-GAG) metabolism” (Fig. 2d ii). In the Naïve-EVs group, in terms of GOBP, the DEPs were related mainly to “cellular process”, and to several related organization processes (e.g. “extracellular matrix and structure” and “external encapsulating structure”) (Fig. 2e i). In terms of GOMF, the DEPs were related to several function terms, of which those related to the bindings (e.g. “carbohydrate derivative”, “cell adhesion molecule” and “calcium ion”), and to “structural molecule activity” (Fig. 2e i). Several pathways related to the REAC pathway were identified, including the enriched pathways of "Extracellular matrix organization", "COPI-mediated anterograde transport", "extracellular matrix (ECM) proteoglycans", "homeostasis", as well as the pathways of "ER to Golgi Anterograde Transport" and "Transport to the Golgi and subsequent modification" (Fig. 2e ii).

#### *Osteo-EVs contain proteins involved in several osteogenesis-related biological processes*

We next sought to identify the participation of the unique proteins and DEPs in each EV group in specific relevant and important biological processes involved in osteogenesis or bone healing. The gene names of the unique proteins and DEPs in the Osteo- and Naïve-EVs groups were compared with those of relevant biological processes from QuickGO annotation (https://www.ebi.ac.uk/QuickGO/). Table 1 lists the EV-identified proteins, including both unique proteins and the DEPs, which matched the selected biological processes. A total of 19 unique proteins and 8 DEPs identified in Osteo-EVs are associated with “cell population proliferation”, “cell migration”, “BMP signalling pathway”, “positive regulation of BMP signalling pathway”, and “Notch pathway”. Four unique proteins in Osteo-EVs are involved in “bone mineralization”, two unique proteins are associated with the “WNT signalling pathway” and most of the 19 unique proteins are associated with “cell migration”. However, none of the DEPs found in Osteo-EVs is associated with “bone mineralization” or the “WNT signalling pathway”. Moreover, Table 1 indicates that WNT5A and NOTCH2, two unique proteins found in Osteo-EVs, are involved in several selected biological pathways, according to QuickGO annotation. In contrast, the table demonstrates that all selected biological pathways are associated with the 12 unique proteins found in the Naïve-EVs group. Furthermore, only 5 DEPs in Naïve-EVs are associated with “cell population proliferation” and “cell migration” and none is associated with the “BMP signalling pathway”, “positive regulation of BMP signalling pathway”, “bone mineralization”, “Notch pathway”, or “WNT signalling pathway”. None of the DEPs found in Naïve-EVs was found to be linked to the “BMP signalling pathway”, “positive regulation of BMP signalling pathway”, “bone mineralization”, “Notch pathway” or the “WNT signalling pathway”.

**Table 1:** Unique EV proteins and DEPs in both Osteo- and Naïve-EV groups

GO Name/Term	Matched protein in each EVs category		Osteo-EVs Naïve-EVs	
	Unique (19/137)	DEPs (8/27)	Unique (12/150)	DEPs (5/22)
Cell population proliferation GO:0008283 (176)	2 proteins: WNT5A, NOTCH2	2 proteins: CSF1, TGFB1	3 proteins: STAT3, CTNNB1, CAV1	4 proteins: ITGB1, GNAI2, RAP1B, GNB1
Cell migration GO:0016477 (133)	9 proteins: ARPC5, PDGFRA, WNT5A, ITGBL1, PAFAH1B1, IGFBP6, SDC1, SDC2, CDH2	4 proteins: AXL, SDC4, TGFB1, ATRN	5 proteins: ENG, JUP, CTNNA1, USP9Y, EPHA2	2 proteins: ITGB1, RRAS
BMP signaling pathway GO:0030509 (91)	3 proteins: TGFBR3, WNT5A, NOTCH2	2 proteins: MEGF8 TGFB1	2 proteins: ENG, USP9Y	
Positive regulation of BMP signaling pathway GO:0030513 (43)	2 proteins: RNF165, NOTCH2	NUMA1	ENG	
Bone mineralization GO:0030282 (54)	4 proteins: ENPP1, ANKH, MINPP1, ASPN		LOX	
Notch Signaling pathway GO:0061314 (170)	3 proteins: NOTCH2, SUSD5, JAG1	2 proteins: TGFB1, APP	ANXA4	
WNT Signaling pathway GO:0016055 (212)	2 proteins: WNT5A, WNT5B		3 proteins: CTNND1, CTNNB1, CPE	

The table presents the unique EV proteins and DEPs in each group (i.e. corresponding gene names) included in the selected GO-terms obtained from QuickGO. The numbers given in parentheses (left column and in the right column in front of each QuickGO term) represent the total number of proteins specific to each GO term. Only protein symbols identified in Homo-sapiens species were retrieved and duplicate protein names were removed from the QuickGO list and EVs dataset. The numbers given in parentheses (beneath each EV category) represent the matched EV proteins compared to the total number of EV group

### Effect of Osteo- and Naïve-EVs on osteogenic differentiation of hMSC in vitro

#### *Internalization of Osteo- and Naïve-EVs by hMSC and effect on their proliferation and migration *in vitro

**Fig. 3:**
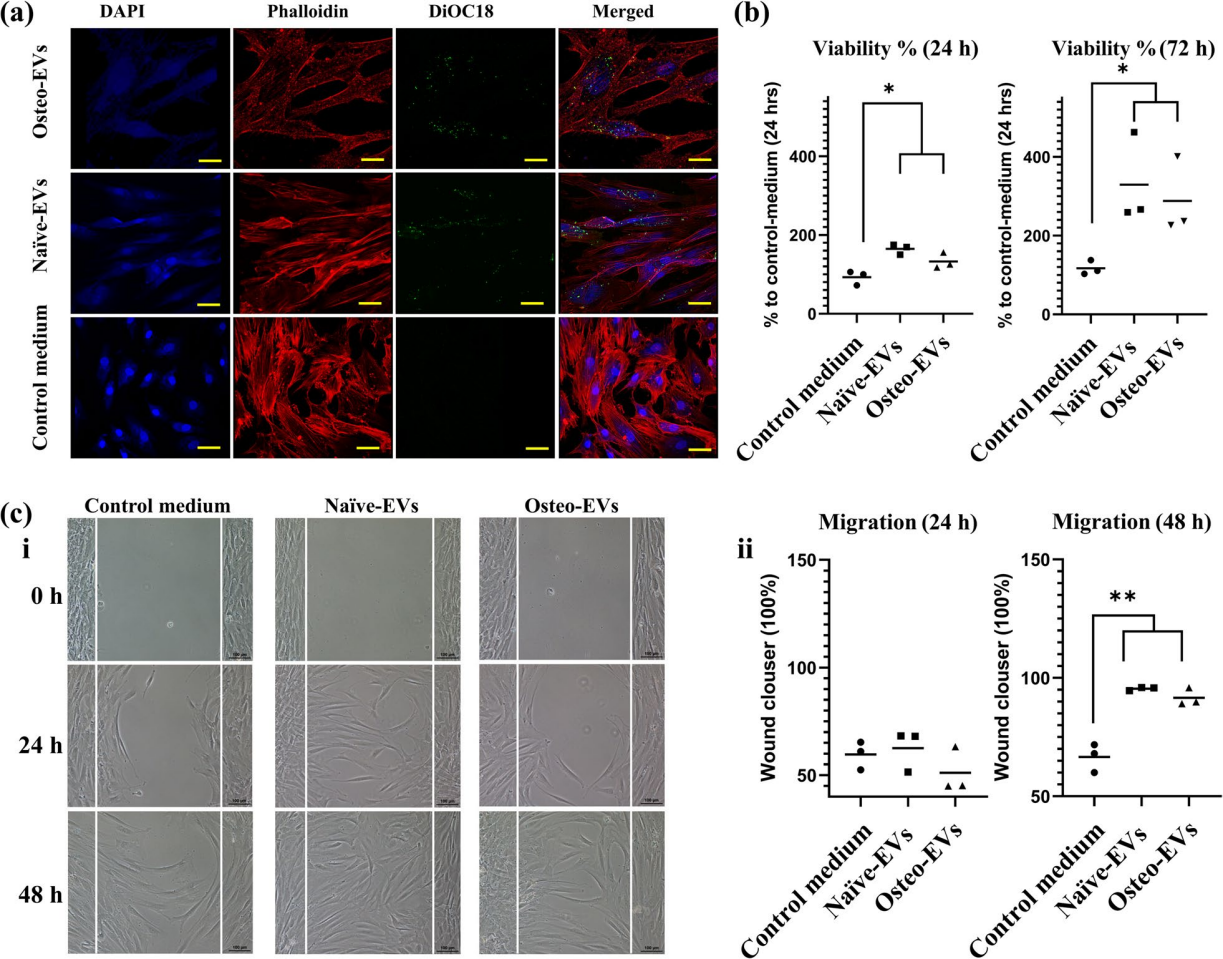
Effects of Osteo- and Naïve-EVs (10 µg/ml each) in cultured hMSC. **a** Representative images from the uptake experiments. Cultured hMSC internalised labelled Osteo- and Naïve-EVs: blue, DAPI-labelled nuclei; red, Phalloidin; green, DiOC18-labelled EVs. **b** Cell proliferation assay indicated that Osteo- and Naïve-EVs stimulate cultured hMSC proliferation after 24 and 72 h compared to the control medium group (BPS and medium). **c i** Representative images from the wound-healing experiments; scale bar 50 µm. Time course of cell migration wound-healing assay for cultured hMSC as the “recipient” cells with the control medium, Naïve- and Osteo-EVs. Scale bar 100 µm. **c ii** Quantification of the wound-healing assay from three independent donors. *p < 0.05, **p < 0.01

To exert their functional effects on target cells, EVs must come into contact with the cell membrane via specific receptors or be internalized by the cells. The data showed that both Osteo- and Naïve-EVs labelled with a green, fluorescent DiOC18 were internalized by hMSC after 48 h of culture compared with the control medium group (Fig. 3a). For cell proliferation, the results indicated that after 24 and 72 h, Osteo- and Naïve-EVs significantly stimulated hMSC proliferation compared with the control medium group, (*p < 0.05) (Fig. 3b). There were no statistically significant differences between the EVs groups. Similarly, for cell migration, the wound-healing assay results revealed a significant increase in cell migration in hMSC treated with Osteo- or Naïve-EVs after 48 h, compared to the control medium group (**p < 0.01, Fig. 3c i and ii). However, no significant differences were observed in cell migration between the hMSC groups treated with EVs, at either 24 or 48 h (Fig. 3c ii).

#### *Effect of Osteo- and Naïve-EVs on osteogenic differentiation potential of hMSC*

**Fig. 4:**
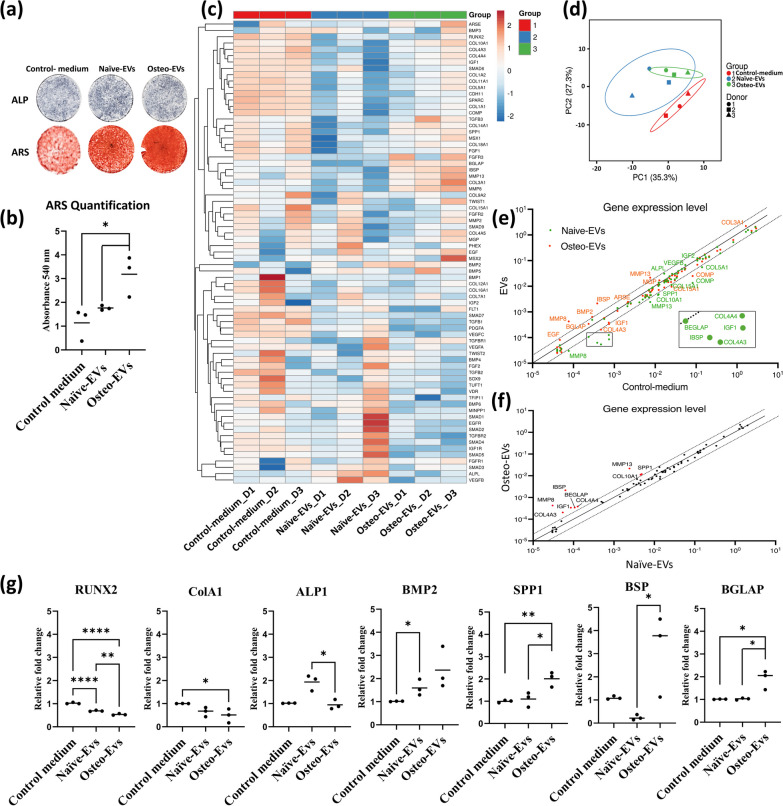
Osteogenic effects of Osteo- and Naïve-EVs in cultured hMSC. **a** Representative images of ALP after 14 days. **b** Representative images of ARS after 21 days. Matrix mineralization was greater after Osteo-EVs treatment than after treatment with the Naïve-EVs and control medium. Quantification of the ARS experiment from three independent donors indicated that Osteo-EVs enhanced matrix mineralization of hMSC after 21 days. **c** Osteogenic gene expression array heatmap among the three donors of each group: control-medium, Naïve- and Osteo-EVs, after 14 days. **d** Prediction ellipses showed a new observation of donors from the same group falling within the prediction ellipse (confidence interval 95%). **e** Gene expression level (fold change) between the control medium and EV groups showed upregulation of several genes related to osteogenesis in the EV groups. Genes with red colours are identified in the Osteo-EVs and those with green colours are identified in the Naïve-EVs; green boxes refer to the downregulated genes in the Naïve-EVs **f** Gene expression level (relative fold change) between the Naïve- and Osteo-EVs showed upregulation of several genes related to osteogenesis in the Osteo-EVs group. **g** Validation of gene array findings with RT-qPCR: Osteo-EVs triggered higher mRNA expression levels of osteogenesis-related markers SPP1, BSP and BGLAP. *p < 0.05, **p < 0.01, ****p < 0.0001

To compare the functional roles of Osteo- and Naïve-EVs in osteogenic differentiation, the hMSC was cultured in OM containing Osteo- or Naïve-EVs (10 µg/ml each), or an equal volume of PBS as a control, in a series of in vitro osteogenic differentiation-related assays. After 14 days, there was a slight increase in ALP in the hMSC treated with Naïve-EVs compared with the Osteo-EVs and control medium groups, as shown in Fig. 4a. After 21 days, ARS staining disclosed significantly greater formation of mineralized matrix in hMSC treated with Osteo-EVs than with Naïve-EVs and control medium (*p < 0.5) (Fig. 4b). To elucidate the genes underlying these effects, we examined the expression of mRNA encoding putative osteogenic differentiation markers, bone extracellular matrices, and molecules involved in TGF/BMP signaling pathways in hMSC, after 14 days of treatment, using a custom-made gene array (87 genes). Of these, 73 genes were detected in all samples and thus considered for further analysis, as shown on the heatmap (Fig. 4c). As shown in the prediction ellipses (Fig. 4d), analysis of gene expression across donors revealed that hMSC donors treated with Osteo-EVs exhibited the lowest variation, followed by the control medium and Naïve-EVs. In the hMSC treated with Osteo-EVs, four genes, namely MMP8, BMP2, IBSP, and MMP13, showed greater than twofold upregulation compared with the hMSC treated with control medium, whereas the Naïve-EVs group showed a trend towards downregulation of osteogenic gene expression (Fig. 4e). It is of interest to note that compared with the hMSC treated with Naïve-EVs, those treated with Osteo-EVs showed a trend towards upregulation of IBSP, MMP8, MMP13, BGLAP, SPP1, IGF1, COL4A3, COL4A4 and COL10A1, (Fig. 4f). In contrast, the hMSC treated with Naïve-EVs exhibited higher ALP expression than those treated with Osteo-EVs (Fig. 4f). RT-qPCR was used to verify the mRNA expression levels of RUNX2, Col1a2, ALP, BMP-2, SPP1, BSP, and BGLAP (Fig. 4g). The mRNA expression of RUNX2 was significantly downregulated in hMSC treated with EVs compared with the control medium (****p < 0.0001) and it was significantly downregulated in the hMSC treated with Osteo-EVs compared with those treated with Naïve-EVs (**p < 0.01). The mRNA expression level of Col1a2 was significantly downregulated only in the hMSC treated with Osteo-EVs, compared with the control medium (*p < 0.05). The mRNA expression of ALP was significantly higher in the hMSC treated with Naïve- than Osteo-EVs (*p < 0.05). Although the mRNA expression level of BMP2 was upregulated in the hMSC treated with Osteo-EV, the only significant level of expression was found in the hMSC treated with the Naïve-EVs compared with the control medium (*p < 0.05). The mRNA expression level of SPP1 was significantly higher in the hMSC treated with Osteo-EVs than in those treated with the control medium and Naïve-EVs (*p < 0.05, and **p < 0.01, respectively). The mRNA expression of BSP was significantly upregulated in the hMSC treated with Osteo-EVs compared with those treated with the Naïve-EVs (*p < 0.05). Similarly, the mRNA expression level of BGLAP was significantly upregulated in the hMSC treated with Osteo-EVs compared with those treated with control medium and Naïve-EVs (*p < 0.05) (Fig. 4g). Based on these in vitro findings, our next step was to conduct a preliminary investigation of the efficacy of Osteo-EVs in repairing bone defects in vivo.

### In vivo osteogenic activity of Osteo-EVs

#### *Adherence of Osteo-EVs to the collagen membrane and uptake by hMSC*

**Fig. 5:**
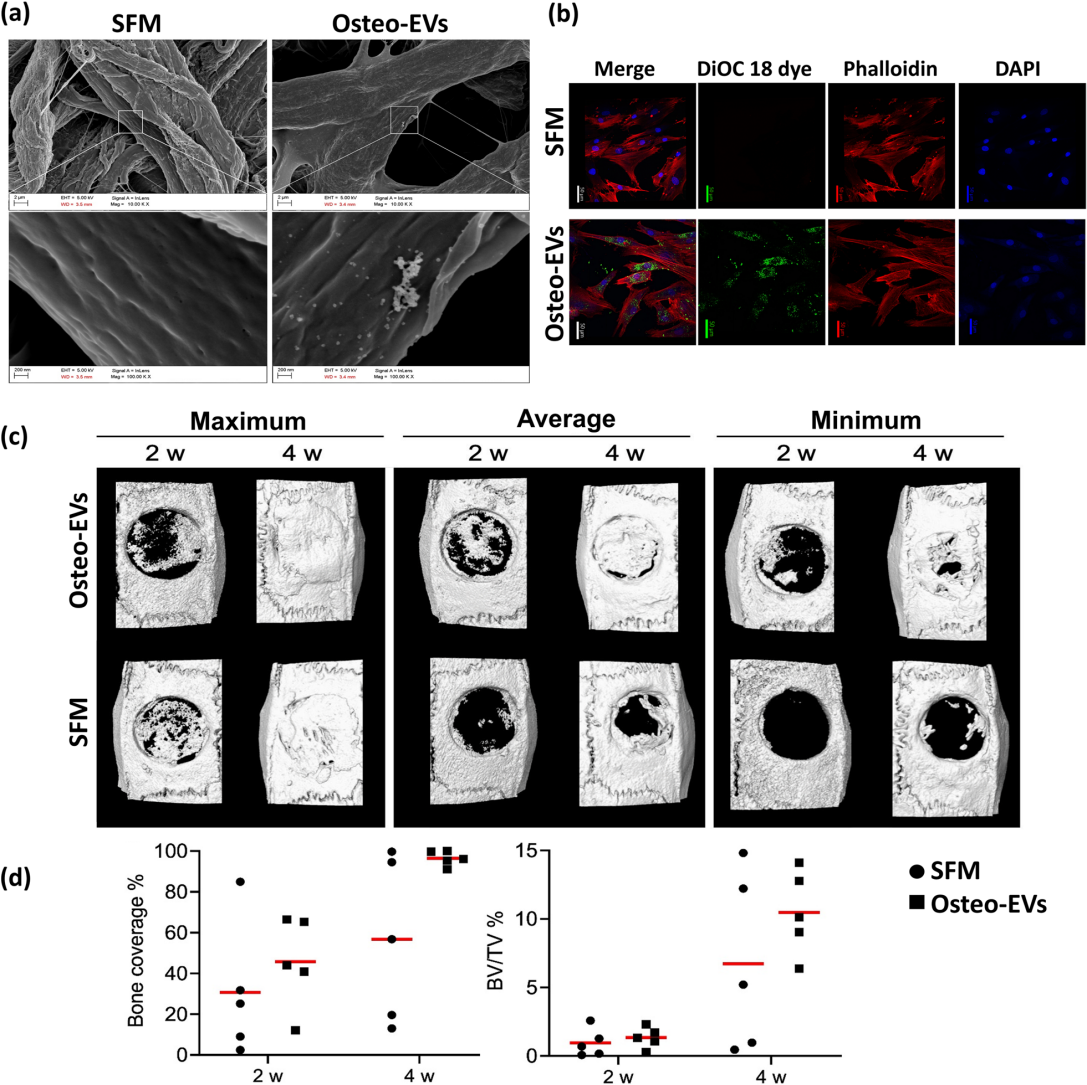
Characterization of Osteo-EVs and MEMs in vitro and in vivo*.* **a** Representative SEM images of the MEM loaded with Osteo-EVs, compared with MEM loaded with only SFM. Scale bars, top: 2 µm, bottom: 200 nm with an average size of 61.47 nm (± 17.47 nm). **b** Representative images from the uptake experiments. Labelled Osteo-EVs were released from the MEM and entered targeted cultured hMSC after 48 h. MEM loaded with SFM served as a control. Blue, DAPI-labelled nuclei; red, Phalloidin; green, DiOC18-dye; scale bar 50 µm. **c** Representative reconstructed µCT images after 2 (in vivo) and 4 weeks (ex vivo) showing maximum, average, and minimum bone formation in Osteo-EVs and SFM groups. **d** Quantification of bone coverage (%) and bone volume per tissue volume (BV/TV %) in SFM and Osteo-EVs groups

SEM showed that the Osteo-EVs (2.7 × 10^10^ particles ≈ 50 µg/MEM) were well distributed over the MEM (Fig. 5a) with an average size of 61.47 nm (± 17.47 nm). To detect the release of Osteo-EVs from the MEM scaffolds and uptake by cultured hMSC, DiOC18 was used to label Osteo-EVs (10 µg/MEM). Dragonfly microscopy of the specimens, after 48 h of incubation, revealed the presence of DiOC18-labelled Osteo-EVs in the cytoplasm and perinuclear regions of hMSC (Fig. 5b), indicating that Osteo-EVs would be released from the MEM scaffold in vivo and then be taken up by resident cells.

#### *Micro-CT analysis of new bone formation*

The bone repair efficacy in vivo of Osteo-EVs loaded onto MEM was assessed in a critical-size rat calvarial bone defect model (Fig. 5c). All animals recovered from surgery and no adverse events were recorded. After 2 weeks, in vivo CT revealed greater coverage of the bone defects in Osteo-EVs (45.76 ± 22.17%) than SFM (30.69 ± 32.59%) (Fig. 5d). A similar trend was observed for BV/TV (1.34 ± 0.75% vs. 0.96 ± 1.03%) (Fig. 5d). At 4 weeks, bone formation was greater after implantation of Osteo-EVs than SFM (Fig. 5c). Although there were no significant differences, the relative coverage of the bone defects was 96.43 ± 3.67% for Osteo-EVs and 56.76 ± 40.50% for the SFM group (Fig. 5d). The level of BV/TV was 10.49 ± 3.06% in Osteo-EVs and 6.73 ± 6.53% in the SFM group, indicating that Osteo-EVs can improve ossification of defects.

#### *Histological and histomorphometric analysis of new bone formation*

**Fig. 6:**
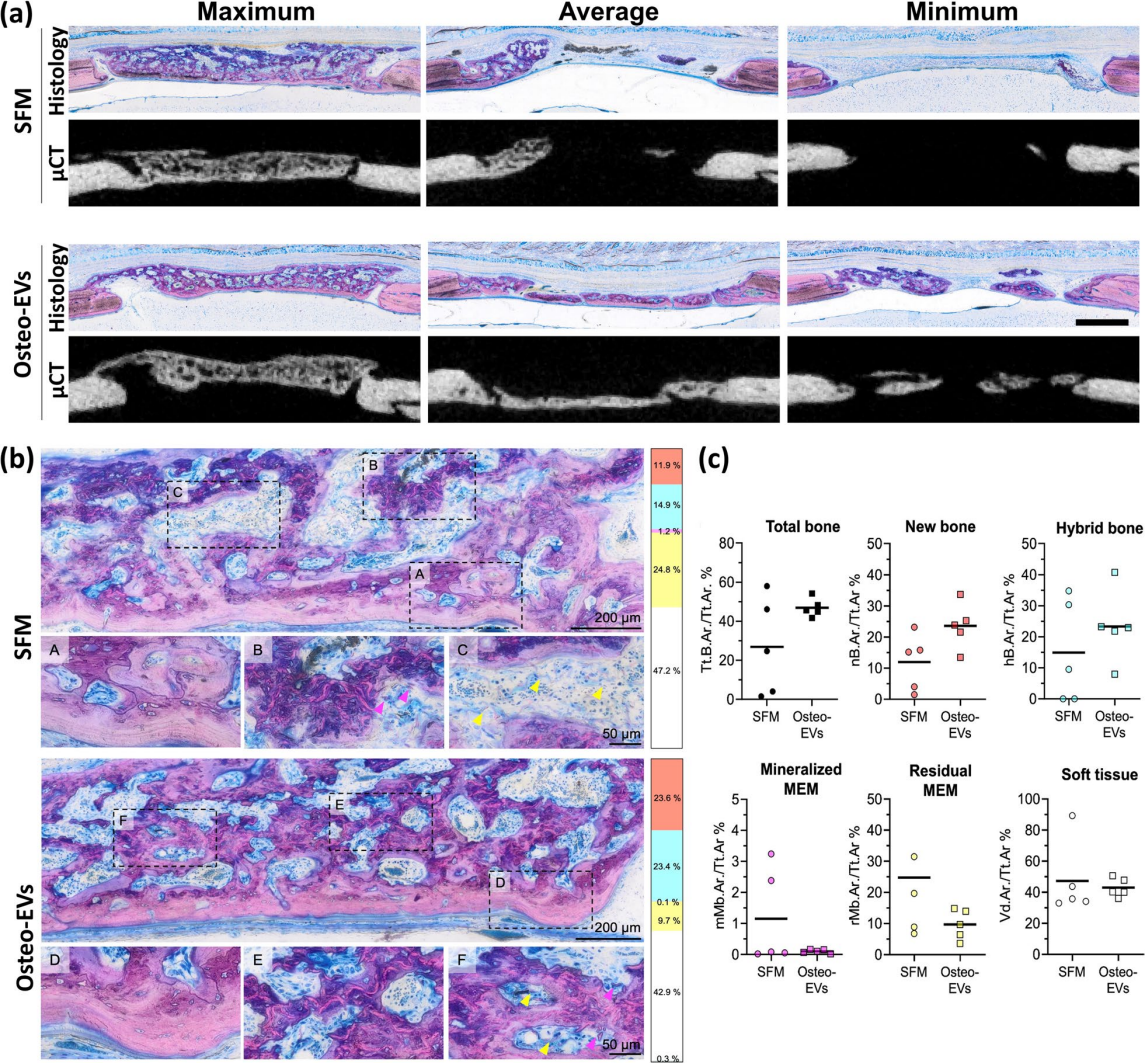
Micro-CT images, histological and histomorphometric analysis of MEM with Osteo-EVs. **a** Representative histological and μCT images of central slices at 4 weeks. Images showing maximum, average, and minimum bone formation in the Osteo-EVs and SFM groups, scale bar: 1 mm. **b** Representative histological images of Levi-Lazko dye staining at higher magnification, showing the different tissues analysed in the Osteo-EVs and SFM groups. The upper panel in each experimental group shows a region of interest with outlined sub-regions, which are enlarged in the lower panel (scale bars: upper 200 um, lower panel 50 um). Each sub-region shows a specific tissue type indicated by letters (A-F). (A and D) new bone (B and E), hybrid bone (C and F) and membrane. Numbers on the side panel indicate relative percentages of new bone for the treatment group (red), hybrid bone (cyan), mineralized membrane fibres (pink), residual unmineralized membrane (yellow) and soft tissue area (white). Yellow arrows; mineralized membrane, pink arrows; residual membrane. **c** Quantification of histomorphometric parameters. Data represent means (*n* = 5). SFM: serum-free medium; MEM: collagen membrane; Total bone (New Bone + Hybrid Bone Area): TtBAr; nB.Ar: New bone area; hB.Ar: Hybrid bone area; mMb.Ar: Mineralized membrane area; rMb.Ar: Residual Membrane area; Vd.Ar: Soft Tissue Area

After 4 weeks, both treatment groups, Osteo-EVs and SFM, exhibited a heterogeneous histological pattern combining the different tissue components after both treatments, as follows: new bone without incorporated MEM fibres, hybrid new bone with incorporated MEM collagen fibres, mineralized MEM fibres, residual MEM, soft tissues, and non-specific mineralization, which could not be attributed to hybrid bone or mineralized MEM fibres (Fig. 6a, b). At the base of the defect towards the dura, i.e. outside the MEM compartment, new bone was typically seen, characterized by well-structured woven bone (dark pink) or surrounded by layers of parallel-fibered bone (light pink) and an osteoid matrix (grey) (Fig. 6b). Adjacent to this newly formed bone an area of hybrid bone was observed, distinguished by immature woven bone, and incorporated collagen fibres from the MEM. Some degree of hybrid bone formation was also found next to the dura, more frequently in the Osteo-EVs/MEM (3/5) than in the SFM/MEM group (1/5). Frequently, hybrid bone was enclosed by new bone without MEM fibres, or in direct contact with residual MEM, indicating that hybrid bone was formed within the collagen MEM: ultimately this could be remodelled to the parallel-fibered bone. Residual MEM was evidently superior to the defect area, partially or completely covering the defect or as free-standing fibres integrated into the soft tissue among the bony networks. The histological evaluation further gave the impression that residual, unmineralized and mineralized fibres could be integrated into the hybrid-stage bone. All samples revealed some mineralized MEM fibres and/or non-specific mineralization. Finally, active bone formation was observed with characteristic osteoblast seams and osteoid (unstained; grey/white) in Osteo-EVs/MEM (5/5), compared with 3/5 in the SFM/MEM group. As shown in Fig. 6c, histomorphometric analysis revealed the presence of these different tissue types in all experimental groups, albeit in different proportions. Quantification of tissues in the central defect area revealed a greater new bone area in the Osteo-EVs/MEM (23.43 ± 7.02%) than in the SFM/MEM group (11.93 ± 9.00%; *p* = 0.054) (Fig. 6c). Quantification of total new bone (new bone + hybrid bone) revealed a non-significant trend (*p* = 0.052) in Osteo-EVs/MEM (46.95 ± 4.68%) compared with SFM/MEM (26.87 ± 25.07%; *p* = 0.110). hMSC treated with Osteo-EVs also exhibited lower areas of both mineralized MEM fibres (0.10 ± 0.57%) and residual MEM (9.70 ± 4.81%), compared with the SFM group, which exhibited mineralized MEM fibres (1.15 ± 1.54%) and residual MEM (24.75 ± 20.52%) (Fig. 6).

## Discussion

This study investigated extracellular vesicles, sourced from both osteogenic and naïve hMSC, with special reference to their potential to promote osteogenesis and bone formation [8]. While EVs from naïve hMSC show positive results, enhancing cell viability, proliferation, and bone formation, EVs derived from osteogenic differentiated hMSC show even more promising therapeutic effects. Nonetheless, it is important not to rely solely on the final outcome, but also to understand the characteristics and content profile of the EVs in question. Therefore, the main aim of this research was to improve understanding of the properties and functions of EVs derived from hMSC during the early stage of osteogenesis: the ultimate objective is to develop more effective acellular therapies for bone tissue disorders.

The current study shows similarities in morphology, size distribution, and expression of common transmembrane markers between Naïve- and Osteo-EVs, i.e. EVs obtained from hMSC during the early stage of osteogenic differentiation. In accordance with previous reports [35], these findings suggest that the brief period of osteogenic differentiation did not significantly alter the overall characteristics of the resultant EVs. MSC-EVs or exosomes exert their therapeutic effects in a content-dependent manner, with proteins, mRNAs and/or microRNAs playing a role in their function [36]. However, the primary focus of the present study was the protein signature of Osteo- and Naïve-EVs, because of their potential therapeutic effects on tissue repair/regeneration. It is important to note that in this study, no profiling of microRNAs was undertaken, despite their potential therapeutic effects [37], because of the limited amount of exosomal RNA available. Although the expression of EV proteins varied among donors, our analysis showed that most of the proteins were common to all. The isolated EVs were also identified by matching the proteins in both EV groups to the Vesiclepedia database. This suggests that the general characteristics of Osteo- and Naïve-EVs are similar and that the inherent cargo of MSC may play a significant role in determining the final EV products [38]. EVs derived from late-stage osteogenic differentiation of hBMSC also shared several proteins with EVs derived from naïve hBMSC [12]. Despite these similarities, we propose that the differentiation state of hMSC after 7 days, *i.e.* at an early stage of osteogenic differentiation, may result in EVs with different protein expressions. It is well documented that the contents of EVs are influenced by the cellular environment and their microenvironments, which in turn can alter the type and concentration of EV proteins and genetic materials such as microRNA [11, 37, 39–43]. This observation is supported by the identification of 187 significantly regulated proteins in response to osteogenic differentiation. Proteins enriched in EVs can modulate various cellular processes, including blood coagulation, apoptosis, extracellular matrix remodelling, and inflammation, all of which are important for tissue repair and regeneration [44]. Moreover, unique proteins related to angiogenesis, apoptosis, inflammation, and extracellular matrix remodelling processes, specifically identified in MSC-EVs suggest that these EVs may play a distinct and critical role in tissue repair and regeneration [44, 45]. In the present study, the upregulated DEPs identified in EVs derived from hMSC during the early stages of osteogenic differentiation were found to be associated with cellular activity, migration, motility, and protein binding. On the other hand, the downregulated proteins, which were upregulated in Naïve-EVs, were found to be involved in cell component organization, metabolism, and binding. In accordance with other reports [46], the findings suggest that osteogenic differentiation of hMSC for 7 days can modify the expression levels of proteins, which are essential for the cellular activity responsible for healing and repair. This was reflected in the involvement of upregulated DEPs identified in Osteo-EVs in pathways related to wound and bone healing, as they regulate critical cellular activities necessary for tissue regeneration and repair [47]. These pathways include extracellular matrix organization, elastic fibre formation, chondroitin sulphate/dermatan sulphate metabolism, Heparan sulphate/heparin (HS-GAG) metabolism, ECM proteoglycans, post-translational protein phosphorylation and modification, metabolism of proteins, platelet degranulation and activation, regulation of insulin-like growth factor (IGF) transport and uptake and response to elevated platelet cytosolic Ca2 + . In contrast, EVs derived from naïve hMSC appear to be involved in pathways related to EVs or exosome formation and biogenesis (see review [48]). For example, the ECM organization pathway is involved in the regulation of cell–matrix interactions, which can affect exosome release and uptake. COPI-mediated anterograde transport, COPI-dependent Golgi-to-ER retrograde traffic, ER to Golgi Anterograde Transport and transport to the Golgi and subsequent modification are all pathways involved in the regulation of protein trafficking and modification within the secretory pathway, which can influence exosome biogenesis and release. Chaperonin-mediated protein is important for proper protein folding, which can affect protein sorting into exosomes and other vesicles. Signaling by Rho GTPases can affect a variety of cellular processes, including vesicle trafficking and exosome release. While the mechanisms responsible for the encapsulation of proteins into EVs and exosomes are not yet fully understood, it is clear that the specific proteins contained within EVs are selectively taken up by cells and their surrounding microenvironments.

During the process of bone repair and remodelling, proper mobilization, proliferation, and activation of MSC are critical. Based on our proteomic analysis, EVs derived from both osteogenic-induced and naïve hMSC can act as nanocarriers for multiple important proteins, can be transferred to recipient cells and cause changes in gene and protein expression affecting the bioactivity of the target cells. For example, the internalization of these EVs may have a positive effect on cell growth and proliferation. We found that the proliferation of cultured hMSC increased significantly after exposure to Osteo- and Naïve-EVs, compared to the control medium group. However, no significant difference in the proliferation of hMSC was observed between Osteo- and Naïve-EVs after 24 or 72 h of treatment. These outcomes were also supported by the QuickGO terms analysis, which identified comparable numbers of proteins involved in the proliferation of the cell population in the different EV groups. Similar results were also achieved when cultured BMSCs were treated with 5 μg/ml EVs derived from chemically induced hMSC at different stages of osteogenic differentiation, compared to those derived from non-induced or naïve MSC [12]. However, the cell proliferation rate increased significantly when the concentration of FBS-depleted EVs was reduced from 10 to 1%, suggesting that FBS concentrations might affect the stimulatory effect of EVs [12]. Based on the QuickGO term analysis, Osteo-EVs were found to contain unique proteins, including WNT5A, WNT5B, TGFB1, NOTCH2, JAG1, and SUSD5. These proteins are known to promote cell proliferation, stem cell renewal and differentiation, and to be critical for bone and cartilage development as well as postnatal bone formation [49–53]. While Naïve-EVs were found to contain potent and unique proteins, including CTNNB1 and STAT3, which not only contribute to cell proliferation but also play vital roles in bone formation [54]. Although further investigation is needed into the roles and underlying pathways of the proteins present in Osteo- and Naïve-EVs, it is evident that they play a synergistic role in promoting not only hMSC proliferation but also other processes necessary for bone formation.

Previous research has shown a stimulating effect on cell migration of EVs or exosomes derived from osteogenic-induced and/or naïve hMSC [55, 56], which is crucial for tissue regeneration [57]. Similarly in this study, treatment with EVs significantly promoted the migration of cultured hMSC after 48 h, compared with the control medium group which did not receive EVs. Despite the identification of a greater number of proteins associated with cell migration in the Osteo-EVs group (Table 1) and the involvement of DEPs in various biological processes related to cell migration and motility, no significant improvement was observed in the migration of cultured hMSC with Osteo-EVs compared with Naïve-EVs. The presence of certain osteogenic proteins in Osteo-EVs may impact the regulation of cell migration ability [58]. Further investigation is warranted into the underlying effects of Osteo-EVs proteins and other factors on cell migration.

The present study also sheds light on the potential therapeutic applications of Osteo-EVs in promoting osteogenic differentiation. In addition to the distinct molecular functions of the upregulated DEPs identified in Osteo-EVs, the QuickGO term analysis also revealed a larger number of proteins involved in the BMP signalling pathway and its positive regulation, Notch signalling pathway and WNT Signaling pathway. In inducing mineralized matrix formation, Osteo-EVs were significantly more effective than Naïve-EVs and positive control medium groups. These findings were supported by gene expression array data and RT-qPCR for osteogenesis-related genes, which showed distinct differences in the effects of Osteo- and Naïve-EVs, particularly for SPP1, BSP, and BGLAP, markers for late osteogenic differentiation and maturation. These findings suggest that higher mRNA levels of late osteogenesis-related genes in the Osteo-EVs treated cells, along with lower mRNA levels and staining of ALP, and higher mineralization nodules, may indicate a more mature stage of differentiation in the Osteo-EV treated cells following the initial proliferation period [59]. Our findings also revealed that the expression of MMP8 and MMP13 genes, which play a critical role in breaking down the triple-helical fibrillar collagen, a crucial component of bone extracellular matrices [60], was significantly upregulated in hMSC treated with Osteo-EVs, compared with Naïve-EVs. Although the pro-osteogenic function of Naïve-EVs was demonstrated by the higher expression of ALP gene in cultured hMSC, the mass spectrometry analysis of DEPs suggests a more general impact of Naïve-EVs on cell metabolism, rather than a specific effect on osteoblastic differentiation and maturation: this may be more related to EV biogenesis and associated processes. Further molecular and genetic investigations are warranted to determine the definitive roles of Osteo- and Naïve-EVs in fundamental cellular events and related pathways.

In the literature, most reports focus on the use of EVs and/or exosomes derived from naïve MSC to enhance cell migration, survival, proliferation, and osteogenic differentiation of cultured MSC in a dose-dependent manner [61]. However, the high doses required for in vivo application may limit their use, because of high costs and unknown side effects [62]. The present study shows that Osteo-EVs contain a range of proteins with bone-targeting and osteoinductive properties and we assumed that a concentration of 50 µg could be beneficial for healing, attracting cells to bone defects and ultimately stimulating new bone formation. Therefore, one aim of the present study was to investigate the potential of Osteo-EVs to promote osteogenesis in order to heal bone defects, while also serving as an easily accessible "off-the-shelf" bone regeneration strategy. In this in vivo investigation, Osteo-EVs were combined with a clinically approved MEM in order to leverage their osteoinductive characteristics. In a recent study, combining MEM with a lyophilized CM obtained from Naïve-MSC resulted in increased new bone formation in rat calvarial defects [19]. In the present study, before in vivo implantation, we first confirmed the binding of the Osteo-EVs to MEM in vitro using SEM. Notably, researchers suggest that the presence of integrins on their membrane gives EVs an affinity for extracellular proteins, especially collagen [20]. The µCT and histological analyses revealed that the combination of Osteo-EVs and MEM scaffolds led to greater and more consistent bone regeneration in critical-sized bone defects than the group treated only with MEM (Fig. 6a). In accordance with a previous investigation [32], implantation of MEM alone into calvarial defects resulted in a diverse histological pattern of formed bone tissue, including hybrid bone tissue with mineralization of the MEM collagen fibres. This can be attributed to the osteoconductive properties of collagen membranes [63], which can potentially promote bone formation, particularly in hybrid bone [64]. In the present study, the hybrid bone was frequently observed to be enclosed by new bone without MEM fibres, or in direct contact with residual MEM, suggesting that the hybrid bone formed within the collagen MEM and may ultimately be remodelled to the parallel-fibered bone. The presence of residual MEM fibres, especially in the SFM/MEM, in the vicinity of hybrid bone also suggests that MEM fibres may play a role in the formation and integration of hybrid bone [19]. In contrast to MEM, the Osteo-EVs in conjunction with MEMs showed limited mineralized fibres, which suggests that the phenomenon of mineralization of existing collagen fibres may not apply to Osteo-EVs. Instead, our study suggests that the facilitation of hybrid bone formation by Osteo-EVs is attributable to their osteogenic protein content, which significantly stimulates the migration, proliferation, osteo-differentiation, and production of ECM proteins by resident cells on the membrane surface. Our results also showed a higher level of active bone formation in the Osteo-EVs/MEM group than in the SFM/MEM group, as evidenced by the presence of characteristic osteoblast seams and osteoid in the Osteo-EVs/MEM group. There was also a small residual membrane area, which may be ascribed to the existence of MMPs in the Osteo-EVs (data not shown) or expressed by resident osteoprogenitor cells, as detected by gene expression array, which are known to facilitate ECM degradation [65]. Together, these factors could create a favourable environment for bone formation: Osteo-EVs show potential for application in bone regeneration and repair [19]. In order to unlock the full potential of Osteo-EVs and other biomaterials for bone regeneration and repair, it is critical to achieve a comprehensive understanding of the mechanisms underlying hybrid bone formation, the effects of Osteo-EVs on biomaterial degradation time and identification of the specific hydrolysis mediators found in Osteo-EVs and their potent roles in this process.

While our study provides valuable insights into the use of Osteo-EVs for bone repair, it is important to acknowledge some limitations. Firstly, a relatively small sample size of only three hMSC donors was used to isolate EVs in vitro: this was to balance the time and expense involved in obtaining EVs and conducting protein analysis, while also ensuring that the sample size was consistent with similar studies of EVs and their protein profiles [65]. Secondly, although LC–MS/MS has high sensitivity and robust resolution for detecting cell secretory proteins, to ensure the reliability and accuracy of the results it is important to confirm the protein changes, using Western Blots or other methods. Thirdly, the in vivo assessment did not include a Naïve-EVs group to confirm the in vitro findings. However, in light of the promising in vitro osteogenic effects of Osteo-EVs, we chose to conduct this in vivo study in a limited number of animals to evaluate the osteogenic potential of a collagen membrane loaded with Osteo-EVs for bone regeneration. This allowed us to determine whether this topic warrants more research, before committing more resources, using larger numbers of animals with similar or other bony defects, such as alveolar bones. In addition, further investigation is necessary to disclose the cellular and molecular mechanisms during earlier stages of bone formation, to reveal potential differences between groups. Finally, further research is essential to determine whether the behaviour and impact of EVs released from biomaterials in vivo can be replicated in cultured cells [8, 66].

## Conclusions

The results show that although EVs derived from naïve hMSC (Naïve-EVs) and early-stage osteogenic differentiated hMSC (Osteo-EVs) share common EV characteristics, Osteo-EVs have a more bone-relevant protein profile. While both types of EVs promote the proliferation and migration of undifferentiated hMSC, only Osteo-EVs significantly enhance their osteogenic differentiation. Finally, the osteogenic potential of Osteo-EVs to enhance GBR was demonstrated in vivo in rat calvaria defects. Together, these findings offer further support for current evidence on the potential of Osteo-EVs as a promising, cell-free approach for bone regeneration. Overall, the study highlights the importance of considering the differentiation stage of MSC when developing strategies for generating MSC-derived EVs for therapeutic bone applications.

### Supplementary Information

**Additional file 1.** Mass spectrometry and gene expression array analysis data.

**Additional file 2: Table 1**. Overview of primers used for RT-qPCR.

## Data Availability

"The mass spectrometry proteomics data have been deposited to the ProteomeXchange Consortium via the PRIDE [67] partner repository, ProteomeCentral Datasets (proteomexchange.org), with the dataset identifier PXD043335". Supplementary data can be obtained by contacting the corresponding author.

## Abbreviations

hMSC: Human mesenchymal stem cells
Naïve-EVs: Undifferentiated hMSC-derived extracellular vesicles
Osteo-EVs: HMSC-derived extracellular vesicles during the early stage of osteogenesis
MEM: Collagen membrane
MSC-CM: Mesenchymal stem cell-conditioned medium
OM: Osteogenic induction medium
SFM: Serum-free medium
SEC: Size-exclusion chromatography
LC-MS/MS: Liquid chromatography with tandem mass spectrometry
DEPs: Differentially expressed proteins
DiOC18: Dioctadecyloxacarbocyanine Perchlorate 18
