# Supplementary material for: Osteogenic human MSC-derived extracellular vesicles regulate MSC activity and osteogenic differentiation and promote bone regeneration in a rat calvarial defect model
Source: Stem Cell Res Ther. 2024 Feb 7;15:33. doi: 10.1186/s13287-024-03639-x (PMC10848378; doi:10.1186/s13287-024-03639-x)
Supplement: Supplementary file 2 — Additional file 2: Table 1. Overview of primers used for RT-qPCR. [file 13287_2024_3639_MOESM2_ESM.docx]

**Additional file 2: Table 1.** **Overview of primers used for RT-qPCR.**

| **Gene** | **Assay ID** |
| --- | --- |
| **GAPDH** | Hs02758991-g1 |
| **RUNX2 (runt-related transcription factor 2)** | Hs01047973-m1 |
| **Col1a2 (collagen type I alpha 2)** | Hs00164004-m1 |
| **BMP-2 (bone morphogenic protein-2)** | Hs00154192-m1 |
| **BSP (bone sialoprotein)** | Hs00960942-m1 |
| **OC (Osteocalcin)** | Hs01587814-g1 |
| **SPP1 (Osteopontin)** | Hs00960942-m1 |
